# Abdominal Ectopic Pregnancy: A Case Report and Literature Review

**DOI:** 10.7759/cureus.84511

**Published:** 2025-05-20

**Authors:** Amani A Eisa, Mohammedelfateh Adam, Shima Alhindi, Afia Azam

**Affiliations:** 1 Obstetrics and Gynaecology, Cork University Maternity Hospital, Cork, IRL; 2 Obstetrics and Gynaecology, Letterkenny University Hospital, Letterkenny, IRL; 3 Reproductive Health, Gender and Reproductive Health and Rights Resource and Advocacy Center (GRACe), Khartoum, SDN; 4 Obstetrics and Gynaecology, Ibrahim Malik Teaching Hospital, Khartoum, SDN

**Keywords:** abdominal pain and ectopic, challenges in diagnosing, ectopic pregnancy, intra-abdominal pregnancy, management of ectopic, suspecting ectopic

## Abstract

Abdominal pregnancy is an uncommon and potentially life-threatening variant of ectopic pregnancy. It involves the implantation of the gestational sac on abdominal organs or the omentum, posing diagnostic and management challenges due to its varied clinical presentations. We present a case of a 34-year-old gravida 7 para 6 woman with progressive lower abdominal pain, initially suspected to have an ovarian ectopic pregnancy based on ultrasound findings. The patient presented with no vaginal bleeding or shoulder tip pain, and her last menstrual period was uncertain due to lactational amenorrhea. A transvaginal ultrasound confirmed an extrauterine pregnancy at 11+2 weeks, with serum beta-human chorionic gonadotropin (BhCG) levels measured at 58818 mIU/ml. During emergency laparoscopy, an abdominal ectopic pregnancy was identified, with placental attachment to the fimbrial end of the fallopian tube and omentum. A salpingectomy was performed while preserving the ovary. This case report highlights the challenges in diagnosing and managing abdominal ectopic pregnancy and emphasises the importance of considering ectopic pregnancy in the differential diagnosis of abdominal pain in women of reproductive age.

## Introduction

Ectopic pregnancy (EP) develops when there is implantation and growth of a pregnancy in any location other than the uterine cavity [1]. EP is classified based on the location, which includes the rudimentary horn, uterine and extrauterine. Cervical and caesarean scar EP is considered uterine EP, while extrauterine EP includes ovarian, abdominal, and tubal, which is further divided into interstitial, isthmic, and ampullary ectopic pregnancies [2].

EP occurs in 11 per 1,000 pregnancies, with an estimated maternal mortality of 0.2 per 1,000 EPs [3]. EP is responsible for 80% of maternal deaths in the first trimester, with non-tubal EP deaths occurring more frequently than tubal pregnancy deaths [4].

According to Long et al., abdominal EP is the rarest form and occurs in less than 1% of all ectopic pregnancies [4]. It refers to a gestational sac implanted in the omentum or abdominal vital organs, which can be primary or secondary. A primary abdominal pregnancy is caused by fertilisation of an ovum in the abdominal cavity, and a secondary abdominal pregnancy is thought to follow early rupture or abortion of a tubal pregnancy or uterine perforation. However, the type of abdominal pregnancy cannot be clinically differentiated. The subsequent criteria have been suggested for the diagnosis of abdominal pregnancy: (i) empty uterine cavity; (ii) absence of a dilated fallopian tube and a complex adnexal mass; (iii) absence of myometrial tissue between the bladder and pregnancy; (iv) a gestational sac surrounded by intestinal tissue and separated by the peritoneum; and (v) free mobility similar to fluctuation of the sac.

The clinical presentation of the EP is variable between minor to significant signs and symptoms, which include abdominal or pelvic pain, shoulder pain, presyncope, syncope, vomiting, diarrhoea, lower urinary tract symptoms, rectal pressure, or pain with defecation [5].

## Case presentation

A 34-year-old gravida 7 para 6 patient with no significant medical history was referred to the emergency department (ED) by her general practitioner (GP) due to complaints of lower abdominal pain. The patient did not report any vaginal bleeding or shoulder tip pain, and her last menstrual period was uncertain due to lactation amenorrhea. She had been experiencing mild abdominal cramping for three days, which progressively worsened with palpation in the right lower quadrant and with movement.

The patient, a Ukrainian national who does not speak English, was accompanied by her husband. This was her seventh pregnancy, with all previous pregnancies being spontaneous and resulting in normal vaginal deliveries, except for gestational diabetes mellitus during her last pregnancy. She denied any history of sexually transmitted infections, pelvic inflammatory disease, intrauterine device use, or abdominal/pelvic surgeries. Additionally, she reported no history of smoking.

Upon presentation to the ED, an initial assessment suggested the possibility of an intrauterine pregnancy; however, an ultrasound examination determined the pregnancy to be extrauterine. The clinical impression was that of an ovarian EP. Communication was initially facilitated via Google Translate (Google LLC, Mountain View, California, United States), followed by the involvement of a professional interpreter. According to the GP's referral, the patient had attended the ED the previous day due to abdominal pain, where blood tests and analgesia were administered, but she was not evaluated by the gynaecology team. Continuing pain prompted her to revisit her GP, who identified mild local peritonism in the right iliac fossa, supra-pubic region, and left iliac fossa.

**Figure 1:**
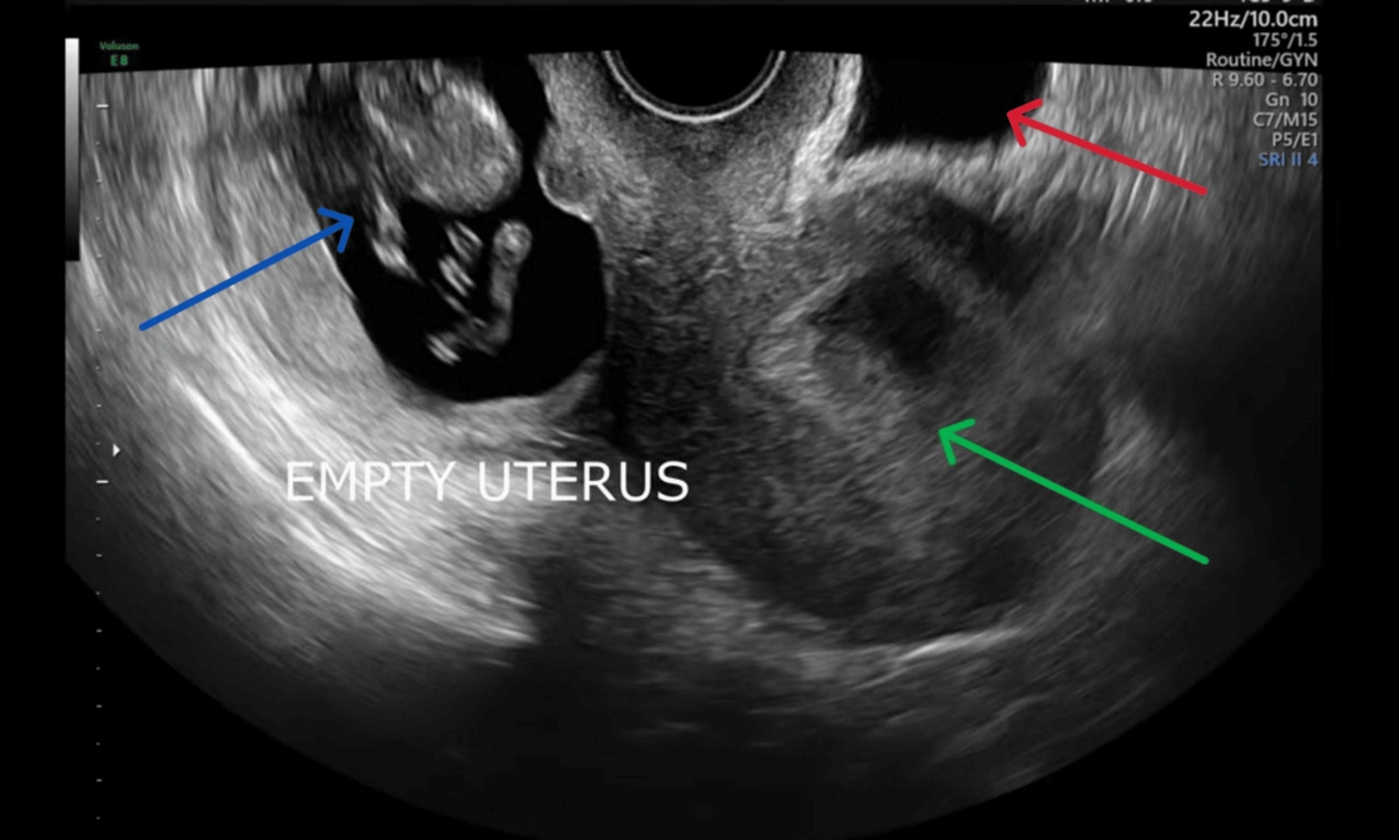
A transvaginal ultrasound shows empty uterus (green arrow) with right ovarian ectopic pregnancy (blue arrow) and the bladder (red arrow)

**Figure 2:**
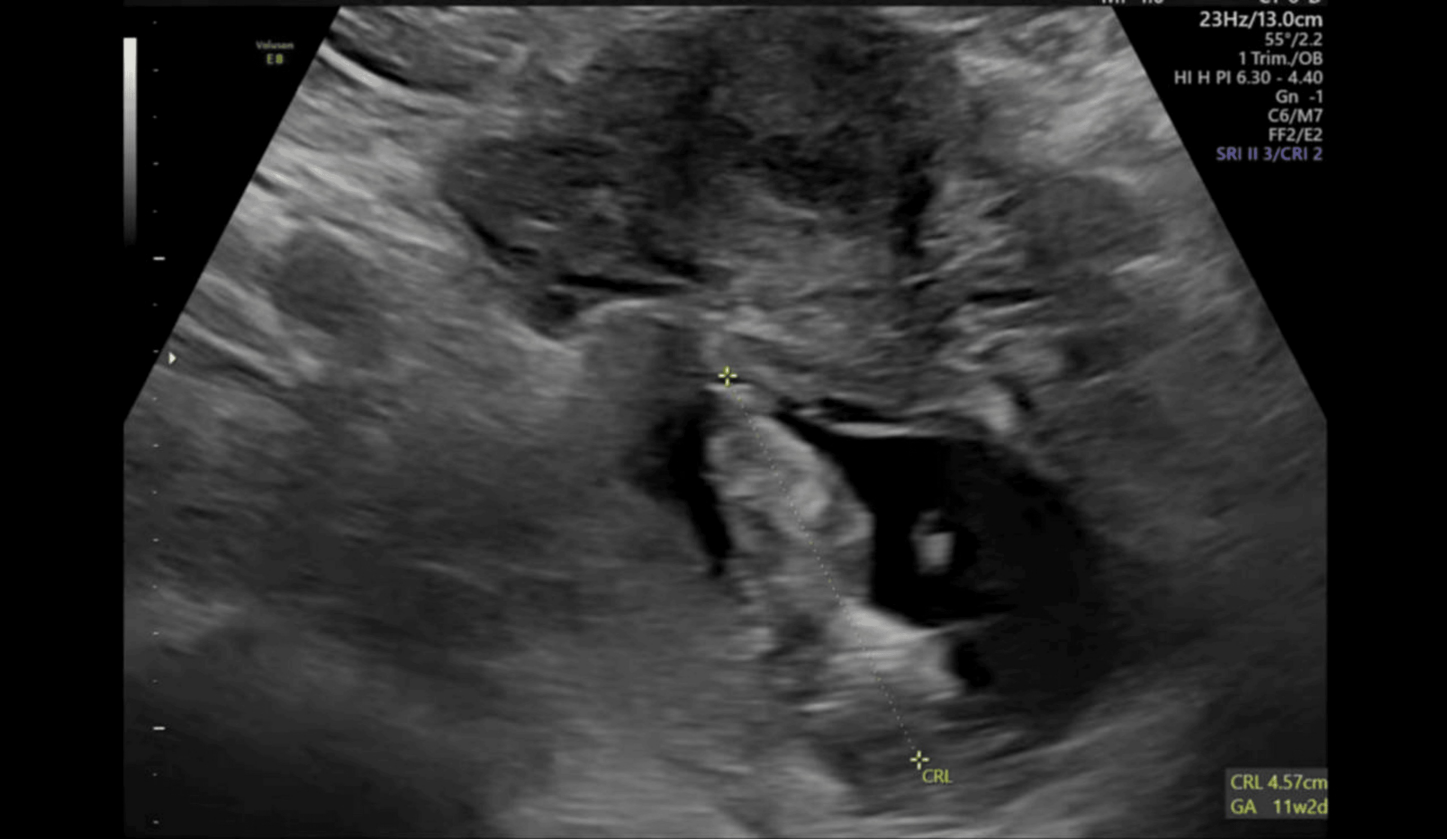
A transvaginal ultrasound Illustrates right ovarian ectopic pregnancy with a crown-rump length (CRL) OF 4.57 cm equivalent to 11 weeks+2 days

A chaperoned examination in the ED indicated mild abdominal tenderness. Serum BhCG was measured at 58818 mIU/ml. A transvaginal ultrasound confirmed the presence of a viable right ovarian EP at 11+2 weeks (Figures 1, 2).

Two intravenous lines were established, and blood samples were collected. The consultant on call obtained informed consent for an urgent laparoscopic salpingectomy, which may have included oophorectomy or laparotomy. Two units of packed red blood cells (RBCs) were cross-matched, and the anaesthetic team evaluated the patient before the procedure.

**Figure 3:**
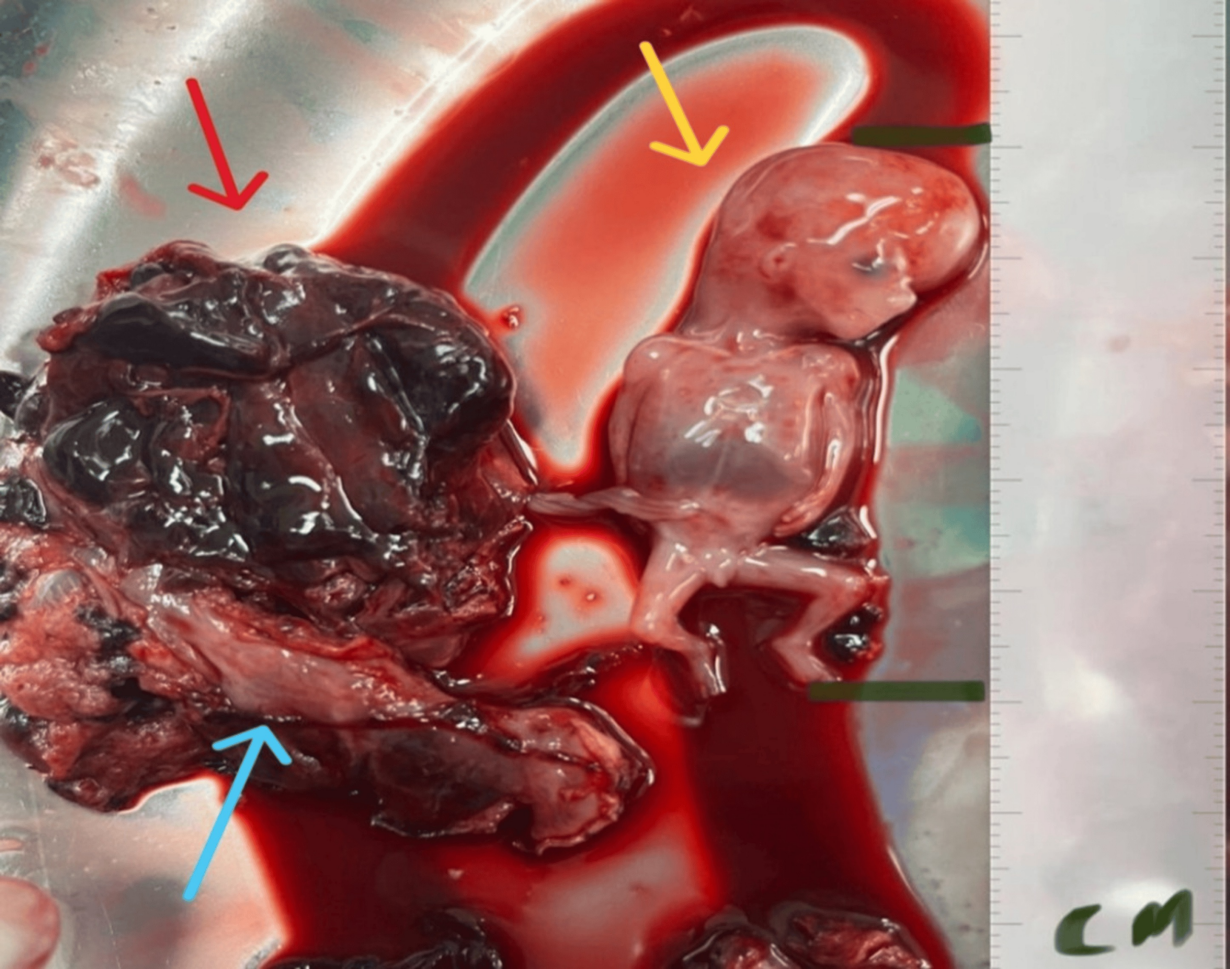
Postoperative findings demonstrate a male foetus (yellow arrow) with the placenta (red arrow) attached to the right fallopian tube (blue arrow)

**Figure 4:**
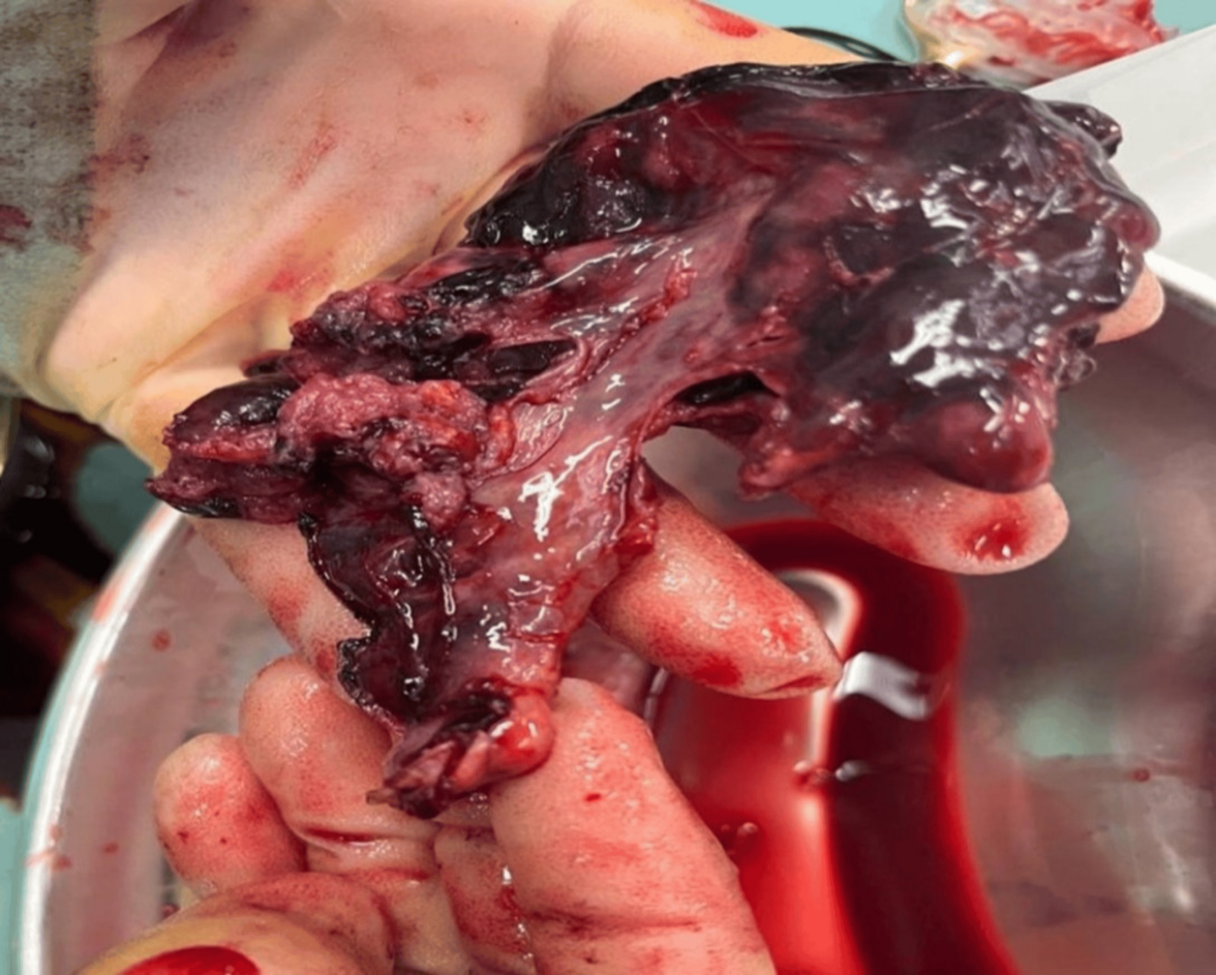
Intact right fallopian tube with placenta attached from outside

During the operation, an abdominal EP was identified. The placenta was noted to be adherent to portions of the fallopian tube and the omentum, primarily infiltrating the fimbriae side of the tube. Consequently, a right salpingectomy was performed while preserving the right ovary. The internal appearance of the tube was normal, with external placental attachment, as seen in Figures 3, 4.

## Discussion

Maintaining a high index of suspicion is critical for the diagnosis of abdominal pregnancy. Unlike tubal EPs, abdominal pregnancies may remain undetected until later stages of gestation. Therefore, this condition is linked to a significant rate of maternal complications. The maternal mortality rate can reach as high as 20%, primarily due to the risk of substantial bleeding resulting from either partial or total placental separation [6].

Abdominal pregnancy presentations vary significantly, with severe abdominal pain reported as one of the most common symptoms [7]. The variability in symptoms may lead to misdiagnosis of abdominal pregnancies. Given the spectrum of clinical presentations, ultrasound examination is typically the primary diagnostic method. It plays a crucial role in the diagnosis of EP [8]. In this case, both transabdominal and transvaginal ultrasounds were utilised in identifying the extrauterine location of the pregnancy. However, transvaginal ultrasound is preferred over transabdominal ultrasound for evaluating ectopic pregnancy, as it offers superior visualisation of the adnexa and uterine cavity [9]. Nonetheless, ultrasound findings may be ambiguous, influenced by the examiner's proficiency and the quality of the equipment, as it happened with this case, initially misdiagnosed as ovarian EP. In such instances, MRI can provide supplementary information for patients requiring a more accurate diagnosis [8].

Management of abdominal EP often requires surgical intervention due to the high risk of haemorrhage [10]. In this case, the decision to proceed with laparoscopy, with the possibility of laparotomy, was appropriate given the clinical findings and the potential risk of haemorrhage. The laparoscopic approach has been proven to be an effective and safe option in managing early EP [11]. The preoperative preparation, including cross-matching of blood and involvement of the anaesthetic team, was vital in managing the potential for intraoperative complications.

## Conclusions

This report highlights the challenges in diagnosing and managing abdominal EP. It underscores the importance of considering EP in the differential diagnosis of abdominal pain in women of reproductive age, the critical role of imaging in diagnosis, and the need for prompt, coordinated surgical intervention to manage this potentially life-threatening condition.

## References

[1] Mullany K, Minneci M, Monjazeb R, C Coiado O (2023). Overview of ectopic pregnancy diagnosis, management, and innovation. Womens Health (Lond).

[2] Kirk E, Ankum P, Jakab A (2020). Terminology for describing normally sited and ectopic pregnancies on ultrasound: ESHRE recommendations for good practice. Hum Reprod Open.

[3] : Guidelines. Ectopic pregnancy and miscarriage: diagnosis and initial management. (2023). National Institute for Health and Care Excellence: Guidelines. Ectopic pregnancy and miscarriage: diagnosis and initial management.. Ectopic Pregnancy and Miscarriage: Diagnosis and Initial Management.

[4] Long Y, Zhu H, Hu Y, Shen L, Fu J, Huang W (2020). Interventions for non-tubal ectopic pregnancy. Cochrane Database Syst Rev.

[5] Sivalingam VN, Duncan WC, Kirk E, Shephard LA, Horne AW (2011). Diagnosis and management of ectopic pregnancy. J Fam Plann Reprod Health Care.

[6] Gorincour G, Boukerrou M (2023). Abdominal ectopic pregnancy. N Engl J Med.

[7] Nkusu Nunyalulendho D, Einterz EM (2008). Advanced abdominal pregnancy: case report and review of 163 cases reported since 1946. Rural Remote Health.

[8] Saquib S, Mohammed Talha WE (2018). Successful management of abdominal pregnancy: two case reports. Oman Med J.

[9] Cohen JM, Weinreb JC, Lowe TW, Brown C (1985). MR imaging of a viable full-term abdominal pregnancy. AJR Am J Roentgenol.

[10] George R, Powers E, Gunby R (2021). Abdominal ectopic pregnancy. Proc (Bayl Univ Med Cent).

[11] Morita Y, Tsutsumi O, Kuramochi K, Momoeda M, Yoshikawa H, Taketani Y (1996). Successful laparoscopic management of primary abdominal pregnancy. Hum Reprod.

